# Do Explicit Estimates of Angular Declination Become Ungrounded in the Presence of a Ground Plane?

**DOI:** 10.1177/2041669518808536

**Published:** 2018-10-29

**Authors:** Umi Keezing, Frank H. Durgin

**Affiliations:** Department of Psychology, Swarthmore College, PA, USA

**Keywords:** distance perception, angular declination, magnitude estimation

## Abstract

In a series of seven experiments (total *N* = 220), it is shown that explicit angular declination judgments are influenced by the presence of a ground plane in the background. This is of theoretical importance because it bears on the interpretation of the relationship between angular declination and perceived distance on a ground plane. Explicit estimates of ground distance are consistent with a simple 1.5 gain in the underlying perceived angular declination function. The experiments show that, in general, functions of estimates of perceived angular declination have a slope of 1.5, but that an additional intercept can often be observed as a result of incorporating changes in ground distance into reports of changes in angular declination. By varying the background context, a variety of functions were observed that are consistent with this contamination hypothesis.

Might the primary parameters of our perceptual experience be mostly hidden from us? For example, the map of retinal disparities in primary visual cortex normally appears as depth in phenomenological awareness, perceived color is based on the relative activities of the cones in the retina, and cortical sensitivity to spatio-temporal correlation underlies our ability to perceive motion. In this article, we consider the relationship between perceived distance along the ground and what seems to be the underlying parameter of angular declination (i.e., angular direction, in the sagittal plane to the point of ground contact, relative to straight ahead; Ooi, Wu, & He, 2001; Sedgwick, 1983). Wallach and O'Leary (1982; see also Williams & Durgin, 2015) used optical distortions to demonstrate that manipulating the perceived direction to an object on the ground affected its perceived distance. Other labs have confirmed a close relationship between angular declination and perceived height and distance (e.g., Gajewski, Philbeck, Pothier, & Chichka, 2010; Gajewski, Wallin, & Philbeck, 2014; Messing & Durgin, 2005; Ooi et al., 2001; Tarampi, Creem-Regehr, & Thompson, 2010). Inasmuch as it is a source of distance information, angular declination logically ought to be primary, but in this article, we will show that there are dissociations between judgments of declination and of distance. We will argue that these dissociations may reflect contamination of explicit angle judgments by distance information. That is, even if early encodings of angular declination information are used to compute ground distance in locomotor space, explicit (conscious) reports of angular separation may become contaminated by perceived depth separations and become imperfectly reflective of the early encodings.

In previous work (e.g., Durgin & Li, 2011), we have shown that there are related biases in reports of distance and reports of angular direction. When asked to verbally estimate the distance to an object on the ground in locomotor space, people typically give numeric values that underestimate the true distance by a factor of about 0.7 (e.g., Foley, Ribeiro-Filho, & Da Silva, 2004; Kelly, Loomis, & Beall, 2004). This systematic underestimation has sometimes been attributed to flaws in the estimation process itself (Loomis & Philbeck, 2008) or a failed scaling of length in explicit measures. But people are much better at judging the heights of objects of a few meters than they are at judging similar distances (e.g., Durgin, Leonard-Solis, Masters, Schmelz, & Li, 2012), which suggests that ground distance underestimation reflects a genuine perceptual bias. Others have suggested that there is simply a compression of visual space in depth (e.g., Wagner, 1985). However, there is a close quantitative correspondence between the observed underestimation of distance and the observed overestimation (i.e., 1.5 gain) in angular declination judgments (e.g., Li & Durgin, 2012): Across many experiments, both explicit and implicit judgments of visual direction have suggested that perceived angular declination is exaggerated with a gain of 1.5 (i.e., angular expansion; Durgin & Li, 2011; Klein, Li, & Durgin, 2016; Li et al., 2013). Given that egocentric distance to an object on level ground can be estimated from knowing one's eye-height and the angular declination to the object (Ooi et al., 2001; Wallach & O'Leary, 1982), distance underestimation with a gain of about 0.7 is expected if angular declination is exaggerated by 1.5. This is illustrated in Figure 1.

**Figure 1.**
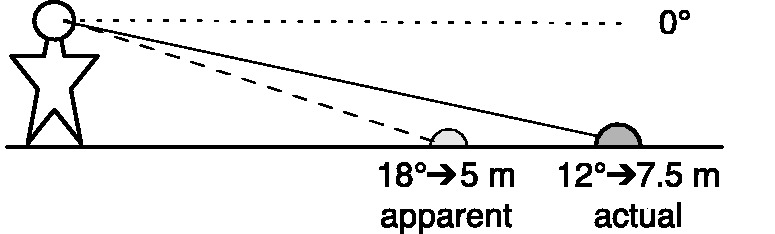
Overestimation of angular declination, relative to straight ahead, by 1.5 predicts underestimation of egocentric distance by about 0.7. If a ball viewed with an angular declination of 12 ° appears to be along an 18 ° line of sight, it should appear to be at a distance of about 5 m (i.e., about 3 eye-heights), much closer than its true 7.5 m distance.

Moreover, nonverbal measures of perceived direction (e.g., positioning oneself so as to make one's egocentric distance from a pole equal to the apparent height of that pole) have consistently been shown to fit the quantitative predictions of the angular expansion model with no free parameters (Li, Phillips, & Durgin, 2011; see also Higashiyama & Ueyama, 1988; Klein et al., 2016). Although angular declination is an egocentric parameter that need not be explicitly represented in allocentric world coordinates (except insofar as it locates the observer within those coordinates), it still makes sense for visual systems to encode and use angular declination information to determine allocentric information such as ground distance, as existing data from many labs have shown (e.g., Ooi et al., 2001, Wallach & O'Leary, 1982).

Finally, it is important to address the worry that action would be adversely affected by a perceptual bias of this sort. Although many have been interpreted as arguing that accurate action implies accurate perception (e.g., Loomis, Da Silva, Fujita, & Fukusima, 1992; Loomis & Philbeck, 2008; Ooi et al., 2001), this is only true up to a scaling factor (Li & Durgin, 2012; Li et al., 2013). Because the visual feedback provided during walking calibrates action (e.g., Rieser, Pick, Ashmead, & Garing, 1995), a near-linear bias in the perception of distance, such as the angular expansion hypothesis implies, would not interfere with the good walking calibration implied by the results of Loomis et al. and Ooi et al. (Durgin, 2014; Li & Durgin, 2012).

Nonetheless, we consider here a concern we have developed about the predictions of the angular expansion hypothesis. In some studies of explicit angular estimation in the real world, a gain of 1.5 is accompanied by a small intercept offset that appears, on the surface, to be inconsistent with the intended explanatory power of the angular expansion theory with respect to ground distance estimation. That is, the measured gain of explicit angular direction estimates in theses studies (∼1.5) corresponds closely with implicit measures of perceived egocentric distance (e.g., Li et al., 2011) assuming a zero intercept in the angular declination function. The intercepts observed in some experiments where a ground plane is present (e.g., 7° in Durgin & Li, 2011, Experiment 1) during explicit estimation tasks therefore appear, at first blush, inconsistent with the theory.

However, it also seems possible that the presence of a ground plane may introduce judgmental biases in the explicit estimation of angular declination (rather than in the early encoding of it). The present study therefore seeks to investigate the estimation of angular declination in the presence or absence of a ground plane. It has recently been observed that explicit judgments of azimuthal direction are larger in the presence of a ground plane (Durgin & Keezing, 2018), and that horizontal–vertical illusions are larger for objects separated in depth on a ground plane (Li & Durgin, 2017). The present investigations were carried out to better understand the possible effects of the ground plane on explicit estimates of angular declination. In the end, we believe that this investigation emphasizes the complexity of measuring perceived declination without undermining the explanatory power of the no-free-parameter quantitative models previously developed in the angular expansion hypothesis (e.g., Li et al., 2011).

In this article, we report all seven studies we have conducted on this topic to date (four are presented as paired experiments). We note this fact in order to inform readers that the experiments reported here are not selected results; they constitute the full body of data that we have collected in our investigation of this topic.

## Experiments 1A and 1B: Testing the Effects of Ground Plane Presence or Absence on Declination Judgments

Our first two experiments use virtual environments to compare judgments of angular declination in the absence or presence of a visual ground plane. In these experiments, we varied whether a visible ground surface was present and whether the target objects were presented along a ground plane surface (whether or not the ground was visible) or suspended in front of a ground plane. To anticipate the main result, the presence of a visible ground plane was found to strongly elevate estimates of angular declination in systematic ways, without fundamentally altering the predicted 1.5 gain. In the absence of a visible ground plane, explicit numeric estimates of angular declination were largely in accord with angular expansion theory (1.5 slope, 0° intercept).

In Experiment 1A, we used a virtual environment that depicted either a rich ground environment or simply a white horizontal line at eye level against a featureless blue background. People made angular declination estimates (relative to straight ahead) to individual balls of fixed retinal size presented as embedded in the ground plane, suspended in the same direction above the ground plane, or suspended in front of a blue background with no ground plane. Our questions were whether the mere presence of a ground plane affected angular declination estimates, and whether it mattered if the targets were in contact with the ground plane.

In Experiment 1B, we added a condition where the balls, even in the absence of a physical ground plane, were presented along an implied ground plane, and we replicated the ground plane contact and no-ground plane conditions with minor changes. First, the ball presented on the ground plane was of constant simulated size, so that its projected size grew smaller with distance. Second, the viewing distance to the ball in the no-ground plane replication was increased to a larger constant distance, to provide greater generalizability.

### Method

#### Participants

Seventy-two undergraduates at Swarthmore College participated for course credit or for payment. In Experiment 1A, 36 participants were divided evenly across the three virtual environments by interleaved assignment. In Experiment 1B, 36 other participants were divided evenly across three other environments by interleaved assignment. The studies were approved by the local institutional review board, and all participants gave informed consent.

#### Design

In both experiments, angular direction estimates were made to balls presented in virtual environments at 11 different angles ranging from 6° to 36° below straight ahead by 3° increments. Because of evidence of strong contaminating influences on judgments across environments (i.e., order effects; Durgin & Keezing, 2018), our analysis only considers the first environment shown to each participant, thus looking at between-subject differences between estimates in the first environment seen.

**Figure 2.**
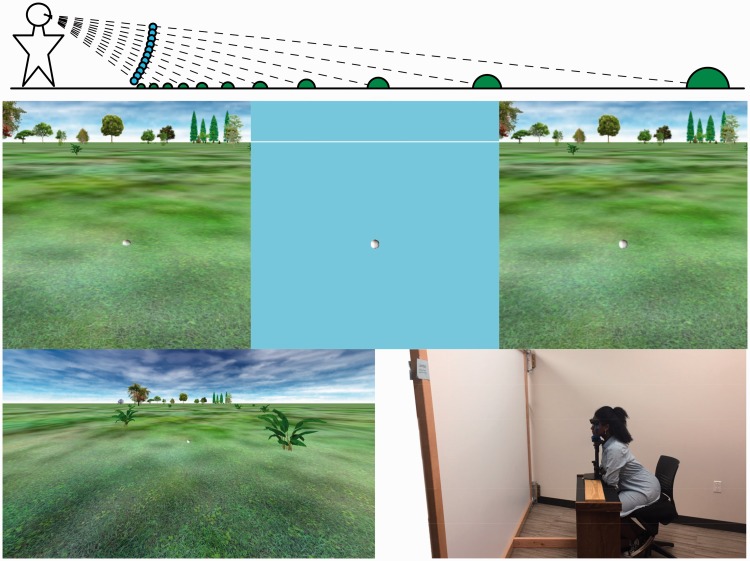
Experiment 1A. The upper panel depicts the set of 11 angular locations (6 ° to 36 °) of both suspended balls (shown here, for clarity, in blue; all 1.9 m from the observer) and their corresponding ground locations and larger ball sizes (shown as green) that project the same horizontal angular extents and directions when embedded in the ground. The three square images across the middle represent details (originally projected at 64 × 64 cm) from individual trials in which a single ball is shown either embedded in the ground (left) like the schematically green balls, or suspended 1.9 m from the observer, like the schematically blue balls, either in the absence of a ground surface (center) or in the presence of a visible ground surface (right). A full screen (256 × 144 cm) view of the latter trial type is shown at lower left. All displays used binocular disparity tuned to the measured interpupillary distance of the observer to specify distance and aligned the virtual ground with the floor of the laboratory in which the observers were seated. The laboratory is shown, with room lights on for the photo, in the lower right panel.

The participants in Experiment 1A estimated the angular declinations to balls in three virtual environments. In one environment, a ground plane was present, and each ball was embedded halfway in the ground, at a ground distance that varied from trial to trial to achieve the intended visual direction (Figure 2, middle left). In the second environment, the scene looked the same (ground plane present), but the balls were suspended above the ground along an imaginary arc around the viewer's head, remaining at a constant (1.9 m) distance from the viewer (Figure 2, middle right). In the remaining environment, the ground plane was absent, and the ball was again suspended in 1 of 11 positions along the same arc (Figure 2, center). A third of the participants made their initial angular judgments in each of the three environments.

**Figure 3.**
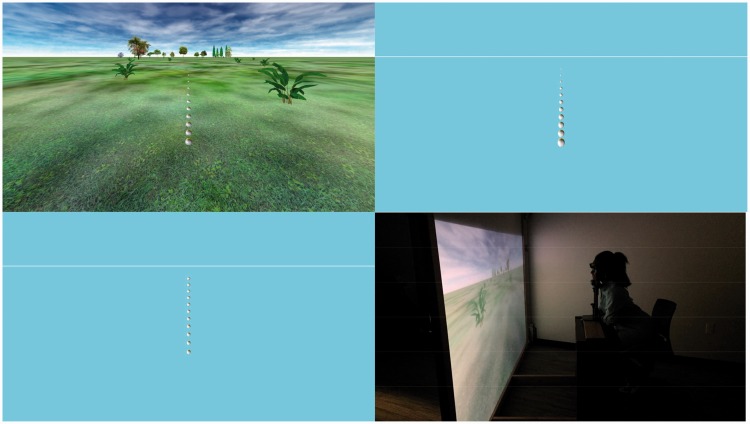
Experiment 1B. The lower right panel shows the physical viewing set-up from a headrest in front of a large back-projection screen while in use. The other three images show all ball positions simultaneously (only one ball was visible during each actual trial) in environments in which the balls were shown either embedded in the ground (upper left), *as if* embedded in the ground in the absence of a ground plane (upper right), or suspended along a virtual arc 3.9 m from the observer in the absence of a ground plane (lower left). All displays were stereoscopic.

The participants in Experiment 1B also estimated angular declination to a ball in one of three virtual environments. In one environment, the ball was embedded in the ground and positioned closer or further from the viewer while the physical ball size was held fixed (Figure 3, top left). In a second environment, the ball was positioned in the same manner, but there was no visible ground plane, so that the ground plane was only implied by ball size and distance (Figure 3, top right). In the remaining environment, the ground plane was again absent, and the ball was suspended in 1 of 11 positions at a constant distance (3.9 m) from the observer along an arc (Figure 3, bottom left). A third of the participants made their initial angular declination judgments in each of the three environments.

In both experiments, the procedure was similar: After an initial practice block in the first environment to be tested, two blocks of 11 experimental trials (randomly ordered) were collected in the primary environment. Ball distance from the viewer ranged from 2.0 m to 11.5 m when the balls were along the visible or implied (i.e., invisible) ground plane. Retinal ball size was constant across all conditions in Experiment 1A (1.5° diameter), meaning that apparent physical ball size grew with distance, as shown in Figure 2, top. Simulated ball size was constant in Experiment 1B (10 cm in diameter), meaning the retinal ball width grew smaller (minimum: 30 arcmin) with increasing distance.

#### Apparatus

Both experiments used stereoscopic virtual scenes (Vizard 5.0; WorldViz, Santa Barbara, CA, USA) projected onto a polarization-retaining back-projection screen (Figures 2 and 3, bottom right). The stereo simulation included a parameter for interpupillary distance (a Shin-Nippon PD-82 was used to assess this) so that the stereoscopic geometry of the scene would be accurate for each observer. The ProPixx projector had a resolution of 1920 × 1080 pixels projected to a size of 2.56 × 1.44 m. An active circular polarizing filter (DepthQ, Lightspeed Design Inc., Bellevue, WA, USA) split the 120 Hz projector signal into 60 Hz left- and right-eye images. Participants viewed the scene through polarizing glasses attached to a fixed forehead rest, such that their eye position was held stationary at a viewing distance of 0.81 m from the screen and an eye-height 1.15 m above the floor, corresponding to the height of the on-screen horizon at a simulated ground distance of 1000 m. The field of view of the projected image extended 53° below straight ahead.

The virtual environments (Figures 2 and 3) always included a visual reference for straight ahead: either the horizon between the ground and the sky or a white line in place of the horizon when the ground plane was absent. The environments with a ground plane also included a cloudy sky and a variety of trees and other plants to enhance the stereoscopic specification of the ground plane in far space.

#### Procedure

The experimenter explained the task of estimating the apparent angular direction to the white ball relative to straight ahead. She informed participants that angular direction could range from 0° (straight ahead) to 180° (directly behind them). They were advised to report direction as they perceived it and to be as precise as possible in their estimates. There was no feedback or other training. On each of the 77 trials (11 practice), participants reported perceived direction in degrees, orally. Their estimate was typed in by the experimenter and presented as text superimposed on the scene, so that participants could confirm that their estimates were recorded accurately. The screen was blanked (using simulated fog) for about 1.5 s between trials after the response was accepted, and before the next trial.

### Results

Angular estimates for the first environments tested (excluding the practice block) were fit with linear mixed-effects models, with visual environments compared between participants.

**Figure 4.**
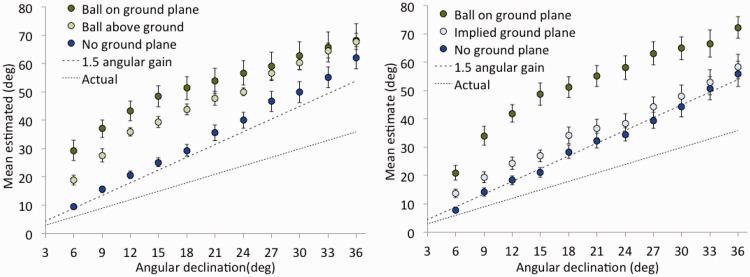
Results of Experiments 1A (left) and 1B (right) as a function of visual direction and environment. The predicted angular gain of 1.5 is shown as a dashed line. Standard errors are shown for each mean.

In Experiment 1A, the overall angular gain in the model did not differ from 1.5 (*M* = 1.53; *SE* = 0.16), and there were no interactions between the effect of direction and environment. The model indicated an intercept of essentially 0 (*M* = −0.37°; *SE* = 2.81°) in the absence of any ground plane but provided evidence that the mere presence of the ground plane (even with balls suspended in near space) increased the intercept to 14.4° (*SE* = 3.97), *t*(33) = 5.13, *p* < .001. When the balls were presented on the ground plane, the intercept increased further still to 27.2°, *t*(33) = 3.23, *p* = .003 (compared with the mere presence condition). Although the graphs in the left panel of Figure 4 suggest some curvature in the direction estimates in the presence of a ground plane, a linear model captures the main feature of the data, which is that the visible ground plane strongly affected the intercept of the angular declination function (but not its overall slope) whether or not the balls were presented on the ground plane.

In Experiment 1B, the overall angular gain in the model (*M* = 1.42; *SE* = 0.12) did not differ from 1.5, and there were again no reliable interactions between the effect of direction and environment. In the absence of any ground plane, the model for balls presented in an arc again indicated an intercept of essentially 0 (*M* = −0.76°; *SE* = 2.2°) but provided evidence that merely presenting the balls in spatial locations consistent with a ground plane increased the intercept, slightly, to 6.35°, *t*(33) = 2.91, *p* = .006. When the same receding balls were presented on the visible ground plane, however, the intercept (21.1°) was reliably higher than when no visible ground plane was present, *t*(33) = 4.80, *p* < .001. The right panel of Figure 4 clearly shows curvature in the angular estimates in the presence of a visible ground plane, similar to the curvature seen in the ground plane conditions of Experiment 1A. Such curvature may indicate a mixture of influences, however, and we will not seek to interpret it closely here. In contrast, a more clearly linear function, with a slightly elevated intercept, was observed when the balls were presented along an invisible ground plane.

**Figure 5.**
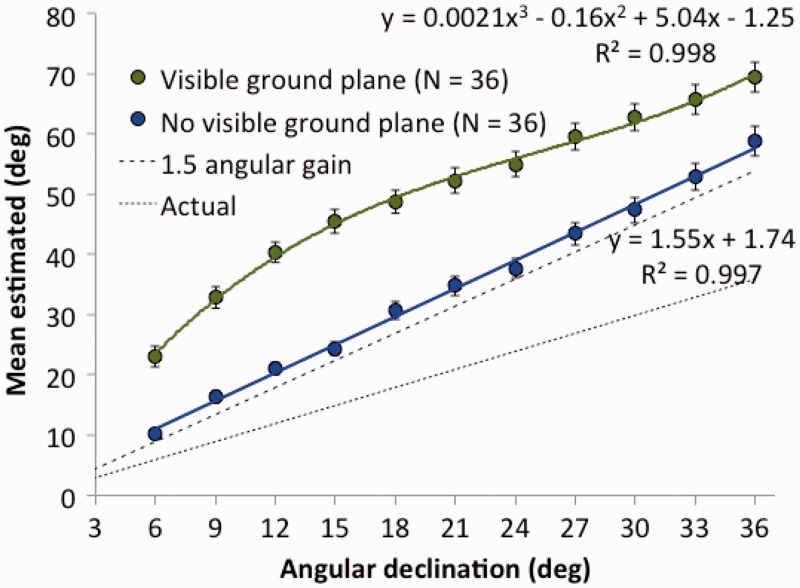
Combined estimates of first environments tested in Experiments 1A and 1B as a function of visual direction and presence or absence of a visible ground plane. The predicted angular gain of 1.5 is shown as a dashed line. Standard errors are shown for each mean.

By combining the results of both experiments together, and considering only whether or not there was a visible ground plane, we can divide the data of all 72 participants into two groups of 36. The resulting means are plotted in Figure 5. It is evident from the figure that the theoretically expected 1.5 angular gain is well represented in the data when there is no ground plane. Conversely, there is clearly both an overall offset (by about 20°) and curvature in the direction estimation function when the ground plane is present. The overall slope, however, remains about 1.5.

### Discussion

Angular expansion theory is predicated on the notion that the angular gain of 1.5 typically found in explicit and implicit measures of perceived declination reflects an important property of an underlying coding scheme. This coding scheme, which can be argued to be informationally efficient because it overrepresents the angular values that are most relevant for the perception of locomotor space, is hypothesized to explain the underestimation of ground distance classically found for this space. However, the present studies show that estimates of angular declination are often elevated much more dramatically when a ground plane is present (though often the slope of the function remains roughly 1.5 overall). This observation is consistent with the possibility that conscious access to absolute angular declination information used to compute distance is somewhat fragile. We suspected that a distortion of the intercept of reported direction might be caused by the salience of the large ground distance to the horizon. If angular coding in early vision is not explicitly preserved in conscious experience in the context of ground surfaces, then estimates of direction certainly could be contaminated by perceived differences in ground distance (i.e., decreasing changes in distance as the balls get equiangularly farther from the horizon).

However, an alternative possibility is that distortions of egocentric distance in virtual environments cause this discrepancy (e.g., Loomis & Knapp, 2003). After all, Durgin and Li (2011) reported an angular gain of 1.5 for estimates made to targets on the ground in an outdoor study. But Durgin and Li did not test on a level ground plane, so the near-horizon directions would not have been associated with particularly large ground distances. Indeed, they intentionally used a sloped ground surface so as to discourage participants from seeking to use inverse geometry to compute angles based on perceived ground distance. Before seeking to further interpret the findings of Experiments 1A and 1B, we conducted an experiment on a relatively flat field outdoors to test whether the results in our virtual environment would be replicated on a level ground plane outdoors.

## Experiments 2A and 2B: Perceived Declination Angle and Perceived Distance Outdoors

Before seeking to interpret Experiments 1A and 1B, we conducted two outdoor experiments in which we first measured perceived direction to six different target distances or directions and later had the same participants make explicit distance estimates to the same distances or directions. This order was used to reduce the likelihood of explicit distance estimation interfering with direction estimation.

### Method

#### Participants

Thirty-two undergraduates at Swarthmore College participated for payment. Sixteen participated in Experiment 2A, and 16 participated in Experiment 2B. The studies were approved by the local institutional review board, and all participants gave informed consent.

#### Design

In both experimental paradigms, participants made estimates of angular declination to multiple positions on a field and later made distance judgments to a similar set of positions. Those in Experiment 2A made angular elevation and distance judgments (separately) to positions that represented a linear range of angular declinations (as in Experiments 1A and 1B). Those in Experiment 2B made them to positions that represented a linear range of distances (as in Durgin & Li, 2011).

**Figure 6.**
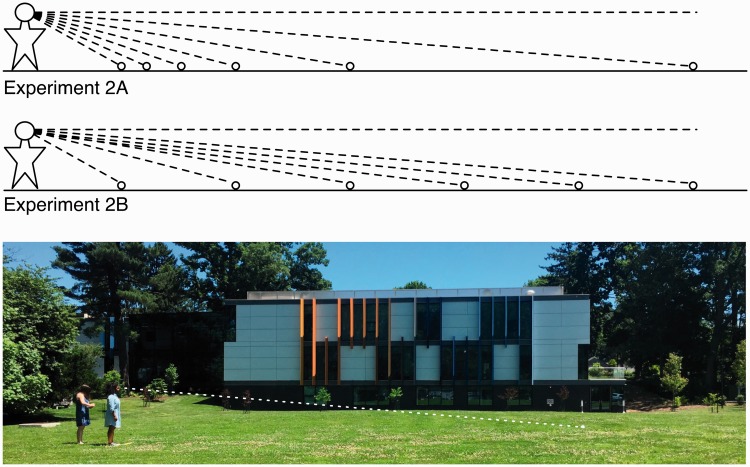
The upper figures show the six equiangular (∼5 °) ball locations used in Experiment 2A and the six equidistant (2.5 m) ball locations used in Experiment 2B. The lower image shows the field location where the studies were conducted. Ball elevation relative to standing location, horizontal ground distance, and eye-height were used to compute the exact angular declination for each target relative to each observer.

Each participant first made judgments of direction, the primary parameter of interest, and then of distance. Six different ball declinations or distances were presented. In Experiment 2A, these ranged at roughly equal angular intervals (of 5°) from 5° to 30° below straight ahead, with some variability in the exact angle presented, depending on the observer's eye-height and the local ground elevation (Figure 6, top); the corresponding distances were 15.0, 7.5, 5.0, 3.8, 3.05, and 2.5 m. In Experiment 2B, the distances were linearly spaced at 2.5 m intervals out to 15 m, producing (on average) angular declinations of 30.6°, 16.1°, 10.8°, 7.8°, 6.0°, and 5.2° (Figure 6, middle panel). Azimuthal direction of the ball from the observer was varied, such that each of the six positions occurred along three different directions in azimuth, to discourage the use of previous ball positions as reference points. The experiments included two blocks of estimates of the six declinations and two blocks of distance estimates to the same angular positions for a total of 24 trials. Within each block, the six declinations or distances were presented in random order and in one of the three azimuthal directions (also specified according to a randomized order).

#### Field apparatus

Experiment 2A took place outdoors in an almost-level field, which was slightly mounded for drainage, with an overall upward slope of about 1° to each of the tested positions (*M* = 1.07°; *SD* = 0.43°). A view of the field is shown in the bottom panel of Figure 6; the colorful psychology building was about 40 m beyond the area used for testing. The field extended about 30 m forward from the viewer, with scattered trees near the far end and a parking lot beyond. A white Styrofoam ball with a diameter of 11.4 cm was placed on the field to mark the direction to be estimated. On each trial, the observer stood on a mat with their feet at a line across the mat, facing the ball. The angular directions to the positions in each study were measured by using a transit level to establish relative elevation and distance. The 6 angular positions were each laid out in three azimuthal directions (along three lines of sight from the observer) for a total of 18 ball positions for each Experiment (12 positions were common to both experiments). The positions were permanently marked by numbered golf tees visible to the experimenter but not to the participants, so that the balls could be placed quickly into positions specified by a randomly ordered data sheet that was individually generated for each participant.

#### Procedure

After measuring their eye-height indoors, the experimenter brought participants to the field and told them to stand on the mat. Participants were given the same instructions regarding direction estimation as in Experiments 1A and 1B. They faced away from the field while the experimenter placed the ball in the correct position for each trial. Once the ball was placed and the experimenter had retreated from the scene, the participant turned to face the field and orally reported the perceived angular declination to the ball. After writing down the estimate, the experimenter instructed the participant to again turn around and then moved the ball to a different position. Once participants had given 12 estimates of angular declination (each declination twice), they were next instructed to estimate distance to the ball along the ground, either in meters or in feet and inches. The trials proceeded as before, though in a new random order, so that the participants completed 12 estimates of perceived distance (each distance twice).

#### Analysis

For each participant, the precise angular declination was computed based on their eye-height and the elevation and ground distance of the ball location in question. Linear mixed-effect regression models were computed for each experiment separately. For plotting purposes, the mean angular declination was used for each of the six nominally distinct angular declinations, and estimation data were collapsed accordingly.

### Results

#### Ground distance estimation

**Figure 7.**
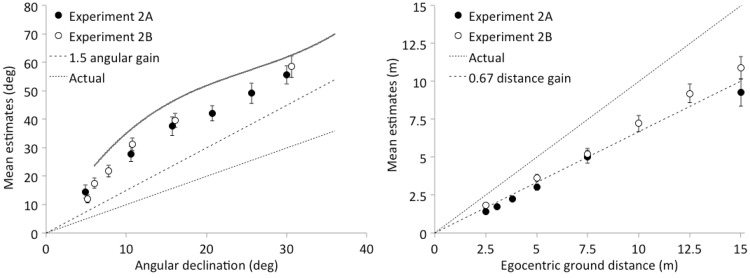
Results of Experiments 2A and 2B. Angular declination estimates (left panel) as a function of actual angular declination are shown with standard errors. The solid line shows the fit to the data from visible ground plane conditions in virtual environments from Experiments 1A and 1B (see Figure 5 earlier). Distance estimates (right panel) as a function of ground distance are shown with standard error bars.

Most studies of explicit egocentric ground distance estimation report underestimation with a gain of about 0.7 or 0.8. In the absence of cognitive corrections based on knowledge (e.g., Durgin et al., 2012), the angular expansion theory (with a gain of 1.5) predicts that ground distance estimates will have a gain of about 0.7 (i.e., 0.67). In Experiment 2A, the slope of the regression model for distance estimation (0.63; *SE* = 0.07) did not differ reliably from 0.67, and the intercept for the model (−0.11 m; *SE* = 0.21 m) did not differ from 0. Similarly, in Experiment 2B, the slope of the regression model (0.73; *SE* = 0.06) did not differ reliably from 0.67, nor did the intercept (−0.11; *SE* = 0.24) differ from 0. A combined model of both sets of data had a slope of 0.69 (*SE* = 0.05) and an intercept of −0.13 m (*SE* = 0.15 m). Thus, as shown in Figure 7 (right panel), in these experiments, the distance estimation data are highly consistent with the general predictions of 1.5 angular scale expansion: Egocentric distance was underestimated by a factor indistinguishable from 0.67.

#### Angular declination estimation

As shown in Figure 7 (left panel), estimates of angular declination resemble the fit line (solid line) from the ground plane visible environments in Experiments 1A and 1B. The linear model slope for angular declination estimates (1.56; *SE* = 0.12) did not differ from 1.5 in Experiment 2A, but there was a nonzero intercept (9.8°; *SE* = 2.8°), *t*(15.1) = 3.55, *p* = .003. Similarly, the model slope (1.73; *SE* = 0.17) did not differ reliably from 1.5 in Experiment 2B, but there was again a nonzero intercept (8.1°; *SE* = 2.2°), *t*(15.0) = 3.68, *p* = .002. A model combining both sets of data had a slope of 1.6 (*SE* = 0.1) and a nonzero intercept (8.9°; *SE* = 1.7°), *t*(30.4) = 5.15, *p* < .001.

### Discussion

In Experiments 1A and 1B, we observed that a visible ground plane seemed to produce a distortion in estimates of angular declination above and beyond the 1.5 gain posited by the angular expansion hypothesis. In the present experiment, we replicated this observation in the real world. That is, the angular gain of verbal estimates still approximated 1.5, but this was accompanied by a reliable nonzero intercept. This occurred even though estimates of ground distance closely conformed to the predictions of the angular expansion hypothesis with a simple gain of 1.5 and no intercept.

Given that the effect of a ground plane on explicit angular declination estimates in virtual environments seems to be replicable in real environments, we next sought an interpretation of the effect. That is, why should the presence of a ground plane produce distance estimates that conform to a 1.5 gain in angular expansion with a zero intercept but produce a nonzero intercept for explicit angular estimation?

One possibility is that the angular information used to produce the distance estimates is not easily accessible for conscious evaluation. Once it has been used for assessing surface layout, angular information may become less available at later perceptual stages of processing, or estimates may be contaminated by the more subjectively salient exocentric properties, such as ground distance. The task of reconstructing angular declination information may therefore be susceptible to biasing by perceived ground distance. When people consciously seek to estimate angular declination relative to straight ahead, it may be that the very extended portion of the ground surface between the horizon and the target biases explicit judgments of angular declination. For example, the ground distance between a 2.5° declination (i.e., an egocentric distance of 36.6 m, assuming a 1.6 m eye-height) and a 5° declination (i.e., a distance of 18.3 m) is much larger than the distance between a 5° declination and a 7.5° declination (i.e., a distance of 12.15 m). Specifically, the ground distance between 2.5° and 5° is three times as extensive as the ground distance between 5° and 7.5°. Large differences in ground distance between equal angular intervals may trigger cognitive comparisons that approximate a logarithmic scaling of explicit reports of declination angles despite the fact that the earlier encoding of angular declination used for evaluating ground distance is not itself affected in this way. On this account, the correspondence of the 1.5 gain with a zero intercept observed in the absence of a ground plane and the 0.7 gain in estimates of ground distance in locomotor space would remain theoretically significant.

In support of the hypothesis that explicit angular estimates are being contaminated by allocentric distances, the curvature observed in angular estimation in the ground plane conditions of our initial studies can be straightened somewhat by plotting estimates against the log of the declination angle. In other words, during the explicit estimation process, it seems that participants may be mistaking large differences in ground distance for large differences in direction. One way to test this distance-contamination hypothesis is to occlude the far ground extents beyond the target balls (even while leaving the rest of the ground plane visible).

## Experiment 3: Occluding Ground Visibility Behind the Targets

Do large ground extents between the target and the horizon cause participants to overestimate angular declination? In Experiment 3, we introduced a large tree trunk in near space, in an attempt to manipulate interference in angular estimation from the extremely large ground extents between target locations near the horizon. We compared a condition where the occluding tree was immediately behind the angular target array area to one where it was off to one side.

### Method

**Figure 8.**
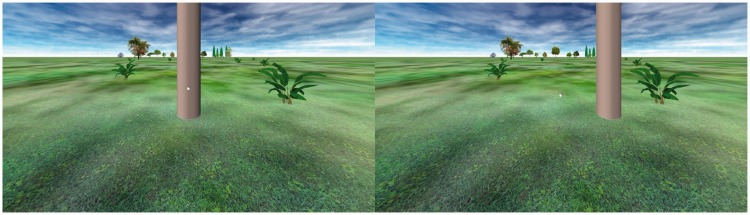
Environments used in Experiment 3. In the experimental condition (left panel), the upper 25 ° of the ground plane is occluded by a large tree trunk. Target balls appear in front of either the trunk or the near grass. In the control condition (right panel), the trunk is moved 1 m off to one side, so the balls appear in front of the ground plane on all trials.

The apparatus, procedure, and environment were essentially identical to those in Experiment 1A when the target balls were suspended in the air in front of a ground plane (1.9 m from the observer), which was clear from the binocular disparity information in the experimental displays. The only visible difference was the addition of a large (0.5 m diameter) tree trunk in the foreground as shown in Figure 8. The only design difference from Experiment 1A was that only two environments were tested. As before, 11 angles (6°–36°) were tested in both environments.

#### Participants

Thirty-six Swarthmore College undergraduates participated for payment. Half of the participants made estimates in the occluded ground condition first; the other half made estimates in the nonoccluded ground condition first. Only first-condition data were analyzed here.

### Results

**Figure 9.**
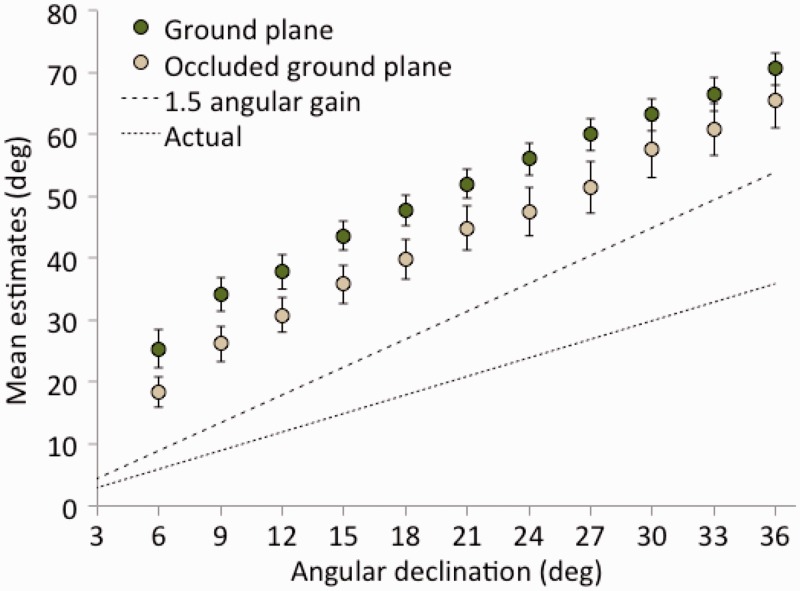
Results of Experiment 3. Mean declination estimates (with standard error bars) as a function of actual declination and ground plane occlusion beyond the target area.

A linear mixed-effects regression on the initial environment data showed that the overall angular gain of declination estimates (1.43; *SE* = 0.12) did not differ from 1.5. When the ground plane behind the targets was not occluded, there was a reliable nonzero intercept (20.6°; *SE* = 3.0°), *t*(34) = 6.74, *p* < .001, as occurred in Experiment 1A. Occluding much of the ground beyond the targets produced a marginally lower intercept (12.2°; *SE* = 4.3°), *t*(34) = 1.95, *p* = .059. Although this is still a nonzero intercept, as Figure 9 shows, the occluded ground plane condition substantially reduced angular estimates across the entire range tested.

### Discussion

Occluding the ground area in the vicinity of the target did seem to reduce the effect of the ground plane on angular estimates, even if it did not eliminate it. Once again, the overall angular gain seems to be 1.5, but there seems to be a pedestal (intercept) added to the judgments. Because the horizon remained the reference for judging angular declination, it may be that participants in both environments (though to differing degrees) were still attending to the distance to the horizon beyond the tree.

## Experiment 4: Manipulating the Distance to the Reference Point

In the previous experiments, the distant horizon (observed or implied) has served as a reference point, and we have hypothesized that the large ground distance between the horizon and the target might have contaminated the estimates of target declination. An alternative hypothesis is that the two-dimensional ground texture produces the distortion on its own. The success of the tree manipulation in Experiment 3 did not clearly distinguish two-dimensional and three-dimensional (3D) accounts because it is unclear what reference point in 3D space participants may have used in the occlusion condition. In the present experiment, we presented a fixed reference ball at the top of a narrow pole that was positioned either very close, in depth, to the farthest ground target or far beyond it. If the distance in depth between the target and the reference is what primarily produces contamination, then angular estimates for the farthest targets (smallest angles) should differ as a function of the whether the 3D distance to the reference ball is small or large (see also Li & Durgin, 2016, for a similar effect in judgments of azimuth).

### Method

**Figure 10.**
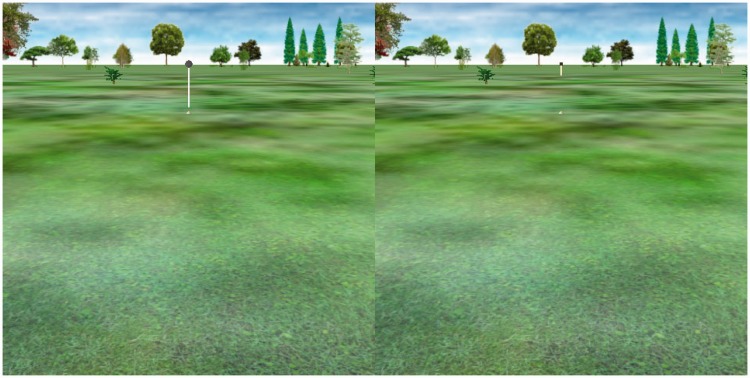
Details of the close reference pole (left) and far reference pole (right) conditions of Experiment 4. The target ball was presented on the ground at various distances; the farthest target (6 ° declination) is shown in each image.

The apparatus and environment were as in the ground condition of Experiment 1B, where the target ball was of constant simulated size and was positioned on the ground (thus decreasing in retinal size at farther positions). As before, the farthest target ball presented (6° declination) was 11.4 m away. The only change was that in the *close* reference condition, there was a pole 12.5 m from the observer holding a reference ball at eye level (just 1.1 m beyond the farthest ball). In the *far* reference condition, the pole was 40 m from the observer (28.6 m beyond the farthest ball). The close and far poles are shown in the left and right panels of Figure 10 along with the farthest target balls. Participants were explicitly instructed to judge the angle between the reference ball on the pole (representing straight ahead) and the ball on the ground.

#### Participants

Forty undergraduates at Swarthmore College participated in the study for course credit.

### Results

**Figure 11.**
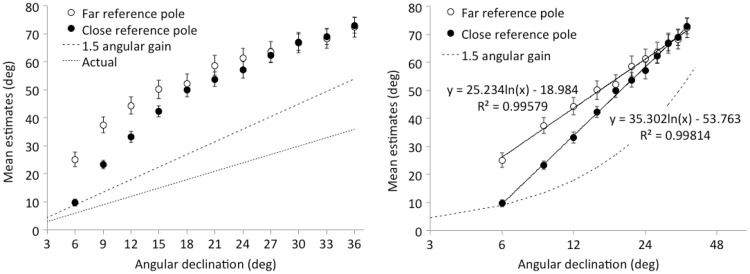
Results of Experiment 4. Angular declination judgments as a function of reference-pole position (near or far) and actual declination (left panel). The right panel plots the same data with a logarithmic abscissa to show that the estimates approximated a linear function of log declination.

As shown in Figure 11, the manipulation of reference distance produced a very different effect than the occlusion manipulation. The right panel of Figure 11 shows that a logarithmic transform of the abscissa works well to linearize the estimation data in this experiment. The *R*^2^ values for the logarithmic fits are 0.996 (far reference pole) and 0.998 (near-reference pole). A linear mixed-effects model was therefore conducted on the declination data using the log-transformed values for the presented angles. This analysis confirmed that the proximity of the reference pole interacted with the effect of angle, *t*(38) = 4.36, *p* < .001. For the ball with the very smallest angular declination (6°), estimates relative to the close reference (*M* = 9.7°; *SE* = 1.2°) did not differ reliably from what is predicted by a 1.5 gain with no intercept (i.e., 9°). The target balls farther from the reference pole depart more and more from a simple 1.5 gain. In contrast, for the far reference, the farthest target position was estimated as 25°, which is reliably higher than the 9.7° observed with the near-reference pole, *t*(38) = 5.30, *p* < .001. Using Bonferonni correction for multiple tests, we found that angular estimates relative to the different reference poles only differed from each other reliably for the smallest three angles.

Can the distance-contamination hypothesis explain why a logarithmic transform linearizes this data? It can because, to a first approximation, logarithmic scaling is consistent with the changing distances of the targets from the reference-pole positions. Rather than a linear increase in angle, as one finds with a blank blue background, there is a decelerating increase in the explicit estimation function as increases in angular declination correspond to smaller and smaller ground intervals in nearer and nearer space. For a target near the depth of the reference, the overestimation ratio is 1.5. But as the distance from the reference increases, the angular estimates grow with a slope greater than 1.5—which might be an effect of larger ground distance changes for these far targets. For nearer targets, however, with declination angles greater than about 18°, the slope of the estimation function returns to about 1.5. This pattern of a decelerating increase conforms pretty well to a logarithmic function.

### Discussion

The near-logarithmic scaling of intervals in the angular estimates observed in this experiment is consistent with the idea that the distance of the target from the reference pole mattered. As distance from the target pole increased, the same angular differences produced less and less difference in estimates given (i.e., the slope of the function flattens out). This pattern is therefore consistent with the hypothesis that the angular declination judgments given in our earlier experiments were contaminated by the perception of ground distance or depth changes and were affected by the apparent distances between, for example, the ground near the horizon and the target. But the clearest evidence of contamination of distance in this experiment concerns the effect of the reference pole on the estimates of the farthest ball: When the reference ball and the farthest ground target were at, essentially, a common distance, the absolute gain of the estimates was 1.5; it was much higher than 1.5 when the reference ball was farther in depth from the target; even then it still showed a slope of 1.5 for relatively high angles. This result is consistent with the introduction of a variable additional offset (essentially a local intercept value) that is determined in part by the perceived ground distance in depth between the target and the reference for straight ahead.

## Experiment 5: Real-World Replication of Reference-Distance Effect on Judgments of Angular Declination

Experiment 5 replicates the close-reference effect in a real environment (and using a lower contrast support pole). The experiment was originally designed to test whether observer orientation would affect explicit estimates of angular declination. A reference ball was presented to remove ambiguities about straight ahead. Previous studies (e.g., Klein et al., 2016) have suggested that viewer orientation has little or no influence on perceived angular direction in elevation relative to a ground plane. However, those studies were principally concerned with angular directions above the ground plane rather than declination down toward the ground. Some of these previous results are quite relevant to our present investigations. For example, Durgin and Keezing (2018; Experiment 2) had sideways observers make estimates of angular elevation above the horizon in a sideways-oriented virtual environment. That is, a ground plane was present, but the balls were presented in an arc against the sky rather than the ground. Their results, in the absence of any ground surface between the balls and the visible horizon, were well modeled by a gain of 1.5 with no intercept. Here, we used balls placed on a real horizontal ground plane but with the observers upright or sideways.

### Method

#### Participants

Forty undergraduates at Swarthmore College participated in the study for payment.

#### Apparatus

The experiment took advantage of large windows in the psychology laboratory building (see Figure 6) overlooking a relatively level ground plane, such that participants could be seated at a chin rest looking out a window instead of into a back-projection screen. Because the first-floor view from our chin rest was elevated 4 m above the outdoor ground surface, the outdoor distances required to represent angles of declination from 36° to 6° ranged from 5.5 m to 38.6 m and were marked on the ground with golf tees. To form a close-reference position, a large Styrofoam ball was held at eye level by a long copper pole (2 cm in diameter) and tripod apparatus at a position 40 m from the building (just beyond the farthest ball position). Three different target ball sizes (4, 9, or 15 cm diameter) were used (depending on the ball location) so that the angular size of the targets was similar at different viewing distances (optical distance ranged from 6.8 m to 38 m; optical ball width ranged from 13 to 31 arcmin, *M* = 21.4 arcmin; *SE* = 1.6 arcmin).

The chin-rest apparatus from the virtual reality experiments was modified to be used at the window. A couch elevated on a custom platform (and with a sideways chin and forehead rest attached) was designed so that sideways participants observed the scene from the same viewing location as upright observers.

#### Design and procedure

Half of the participants made angular declination judgments while upright and half made judgments while lying sideways on the elevated couch. Each participant made judgments of each of 11 declinations from 6° to 36° (by 3° increments) in random order, having been instructed to use the reference ball to establish straight ahead. Between trials, the participants closed their eyes while an assistant outside placed the appropriate target ball in its position for the next trial and then stood out of sight. An experimenter inside the laboratory recorded the participant's estimates and instructed them when to close and open their eyes as the ball was being repositioned.

### Results

**Figure 12.**
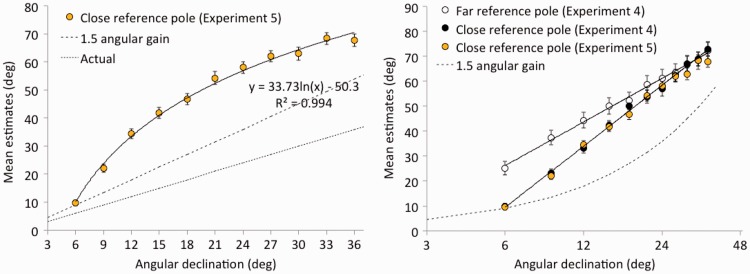
Results of Experiment 5. Estimates of angular declination to balls viewed on a ground plane from a window at an eye-height of 4 m (left panel) are replotted (right panel) with a logarithmic abscissa for comparison with the results of Experiment 4. Standard errors of the mean are shown.

As in Experiment 4, a logarithmic function provided an excellent fit to the estimation data (*R*^2^ = .994), so linear mixed-effects analyses were again carried out on responses with respect to log declination. There was no reliable difference between the estimates of upright and sideways observers, *t*(38) = 1.15, *p* = .257. The mean estimates in the combined upright and sideways conditions are shown in the left panel of Figure 12. As in the near-reference condition of Experiment 4, the mean estimate (*M* = 9.7°; *SE* = 1.0°) for the point nearest the reference pole (i.e., the 6° declination) did not differ from the (9°) prediction of a 1.5 gain angular expansion model. Indeed, in the right panel of Figure 12, the mean estimation data from Experiment 5 are presented alongside the estimation data from Experiment 4. There is essentially no difference between the mean estimates in Experiment 5 and those in the (geometrically similar) close-reference condition of Experiment 4.

### Discussion

In Experiment 4, we observed that placing a reference object (to indicate straight ahead) just beyond the farthest ball position resulted in a normally expanded (1.5) gain for angular declination estimates at that farthest location. This observation was exactly replicated in Experiment 5 despite a variety of differences between the two experimental paradigms. This replication supports the hypothesis that explicit estimates of angular declination relative to straight ahead can be subjected to contamination from depth extents that occupy the region between the reference and the target location. When those depth extents are minimized, as for this farthest target, an angular gain of 1.5, with no additional exaggeration, reemerges.

## General Discussion

The experiments reported here document that the simple 1.5 angular gain in perceived angular declination that has been postulated by the angular expansion hypothesis (e.g., Durgin & Li, 2011) is most easily observed (without any evidence of an intercept) when the distance to the straight-ahead reference is similar to the distance to the target. In Experiments 1A and 1B, we saw that eliminating the visible ground plane was associated with angular estimation functions with gains of 1.5 and zero intercepts. Moreover, similar findings had been obtained when judgments of elevation were recorded against a blue sky above a ground plane (Durgin & Keezing, 2018). In contrast, the shape and slope of the angular estimation functions in the other experiments presented here varied a great deal. In some cases, logarithmic functions seem to provide good fits to the estimation data. This was particularly evident in Experiments 4 and 5, where a near-reference object was used to indicate straight ahead. In other experiments, the functions appear mostly linear but are elevated. This occurred in Experiment 3, where a near tree was used to occlude the far ground extents between the target balls and the horizon.

In general, although the estimation functions produced by participants may reflect a variety of sources of cognitive bias or distortion, we suspect that these more complex angular estimation functions should not be interpreted as uncontaminated measures of the underlying angular parameters that likely contribute to distance perception in motor space. After all, across a broad variety of tasks involving walking (and throwing—Williams & Durgin, 2015), angular declination has been implicated as a powerful source of information concerning ground distance in the range of distances for which declination might be expected to provide useful information.

The simple 1.5 gain posited in the angular expansion hypothesis (and evident in many different implicit and explicit measures; Durgin & Li, 2011) is consistent with the widespread and common underestimation of egocentric ground distance found in many studies of locomotor space, including Experiments 2A and 2B of this article. Given that explicit estimates of angular declination against a blue background in Experiments 1A and 1B also represent a 1.5 gain with a zero (or near-zero) intercept, it seems more parsimonious to assume that the biases that influence explicit estimates of angular declination in the presence of a ground plane are not affecting the underlying angular variables that control our perception of distance.

After all, the estimation of small declination angles is where most of the hypothesized contamination arises. For far (vista) distances, other information besides angular declination is likely relied on much more extensively (see Daum & Hecht, 2009). Even with a hypothesized 1.5 gain in angular declination coding, there may be very little visuo-cognitive precision left to reliably measure 90 m (1.02° of declination) or 120m (0.76°) based on declination information alone, whereas distances like 9m (10.1°) and 12 m (7.6°) can probably be evaluated much more reliably by means of differential declination information.

Indeed, the dissociation between distance underestimation and angular overestimation we observed in Experiments 2A and 2B may reasonably be said to provide important support for the angular expansion hypothesis. If angular estimates in the presence of a ground plane were always perfectly consistent with distance estimates (i.e., showed no intercept), it could be argued that distance compression causes angular overestimation. However, the fact that angular estimates collected in the absence of a visible ground surface correspond to distance estimates in the presence of a visible ground surface shows that a postulated angular expansion with a gain of 1.5 can quantitatively explain ground distance underestimation. Moreover, in nonverbal perceptual tasks in the presence of a ground surface (e.g., Higashiyama & Ueyama, 1988; Li et al., 2011), and a variety of other implicit directions tasks (Durgin & Li, 2011), a 1.5 angular gain is also implied.

Conversely, our data show that distance underestimation does not quantitatively explain explicit angular estimates that are made in the same (ground surface present) context. In other words, the alignment between angular expansion (i.e., with a 0 intercept and a 1.5 gain) and distance underestimation cannot be an artifact of participants using perceived ground distance to estimate declination angle, because they do not typically show a zero intercept in their angular estimates in this circumstance. Thus, both empirically and theoretically, it is reasonable to understand that encoding distortions in declination angle are responsible for the stable underestimation of ground distance in locomotor space to which our actions are normally well calibrated. Such angular expansion represents an informationally efficient coding scheme and is therefore evolutionarily advantageous.

Throughout this article, we have shown how explicit judgments of direction are strongly influenced by information specifying distance. But the relationships we have observed between judgments of distance and judgments of direction indicate that explicit judgments of direction are often inconsistent with those of distance. However, if we assume that there is a stable underlying angular expansion (to which we have limited conscious access), then both the very stable underestimates of distance (explained by angular expansion) and the very unstable estimates of direction (explained by partly confusing 3D separation with angular separation) can be understood as outcomes consistent with the primacy of (expanded) angular variables in computation, but the primacy of distance variables in conscious experience.

In some sense, the spherical coordinate system of the human eye means that direction is *given* in the optic array, whereas distance is not. This is why angular declination is such an important and well-established means for controlling action with respect to ground locations in locomotor space. It is a robust proximal variable that can be extracted even with very poor vision (Tarampi et al., 2010). On the other hand, explicit reports of angular direction are clearly (and systematically) subject to vagaries of the contents of the perceived layout of surfaces and the depth of the reference point, such as have been documented in this study.
